# Phylogeny and divergence of the 100 most common *Salmonella* serovars available in the NCBI Pathogen Detection database

**DOI:** 10.3389/fmicb.2025.1547190

**Published:** 2025-06-13

**Authors:** Linghuan Yang, Hilal Samut, Leonie Kemmerling, Renato Hohl Orsi, Martin Wiedmann, Ruixi Chen, Cristina Resendiz-Moctezuma

**Affiliations:** Department of Food Science, Cornell University, Ithaca, NY, United States

**Keywords:** *Salmonella enterica*, serovar, phylogeny, evolution, divergence, whole-genome sequence, antigen, polyphyletic

## Abstract

Despite the emergence of whole genome sequencing (WGS) for *Salmonella* characterization, serotype assignment remains important as it allows identification of *Salmonella* subgroups that differ in distribution, virulence, and ecology. However, it has been shown that multiple divergent lineages of the same *Salmonella* serovar may have evolved independently multiple times and may present distinct epidemiological characteristics. Previous studies that aimed to identify the phylogeny of certain *Salmonella* serovars often used isolates from specific geographical locations or outbreaks and a small number of isolates to infer the phylogeny. To address these limitations and to advance the understanding of *Salmonella*’s evolutionary patterns, we (i) identified the phylogenetic grouping (i.e., mono-, para-, or polyphyly) of the 100 most common *Salmonella* serovars analyzing 63,204 genomes available in the NCBI Pathogen Detection database, (ii) identified, for each polyphyletic serovar, the lineages that contain the majority of genomes, and (iii) inferred the antigen divergence between the five most common serovars (i.e., *Salmonella* Enteritidis, Typhimurium, Newport, I 4,[5],12:i:-, and Infantis) and their respective closely-related serovars. Among the 100 most common *Salmonella* serovars analyzed, 19 serovars are monophyletic, nine are paraphyletic, and 72 are polyphyletic. In 47 of the 72 polyphyletic serovars, one lineage contains more than 90% of the serovar’s confirmed genomes. Antigen divergence results suggest that serovars Typhimurium and I 4,[5],12:i:- (often referred to as monophasic Typhimurium) have emerged independently of each other multiple times, except for the major I 4,[5],12:i:- lineage, which emerged from the major Typhimurium lineage. Furthermore, divergence in *Salmonella* serovars appears to primarily occur through modifications in the H1 antigen. Hence, this study shows that (i) a much larger number of serovars than previously known are polyphyletic; (ii) serovars previously known to be polyphyletic contain more lineages than previously known; and (iii) many serovars include lineages that only have a few isolates with a given serovar. Our data suggests that, in the age of genomics, molecular serotyping should be combined with other phylogenetically informative approaches to not just assign a serovar but to also indicate the serovar lineage for polyphyletic and paraphyletic serovars.

## 1 Introduction

Salmonellosis, one of the most common foodborne diseases in the world, is caused by bacteria from the genus *Salmonella*, which has two species, *enterica* and *bongori*. *Salmonella enterica* is further divided into six subspecies (i.e., *enterica*, *salamae*, *houtenae*, *arizonae*, *diarizonae*, and *indica*) (Coburn et al., 2007), with at least one study suggesting 11 subspecies (Pearce et al., 2021); however, the status of these five additional subspecies still needs to be confirmed and formally accepted. Within *Salmonella enterica* (*S*.), more than 2,600 serovars have been identified; of these, nearly 1,500 serovars belong to subspecies *enterica* (Issenhuth-Jeanjean et al., 2014). *Salmonella* serovars can be further classified into two groups: typhoidal (i.e., Typhi, Paratyphi A, Paratyphi B, Paratyphi C, and Sendai), and non-typhoidal *Salmonella* (NTS; e.g., Typhimurium, Enteritidis, and Infantis) serovars. While the five typhoidal serovars mainly cause typhoid fever in humans, NTS are usually characterized by gastroenteritis with symptoms including acute abdominal pain, diarrhea, vomiting, and sometimes nausea (Eng et al., 2015). NTS serovars stand out as a large group of pathogens with diverse ecological niches and a notable impact on public health (Cheng et al., 2019). Approximately, 93.8 million human illnesses, and 155,000 deaths are estimated to be caused by NTS serovars annually worldwide (Murray et al., 2012), while two of the typhoidal serovars (i.e., *S.* Typhi and *S.* Paratyphi A) are estimated to cause 25.7 million human illnesses and 178,000 deaths annually worldwide (Kirk et al., 2015).

Within *Salmonella* serovars, some are monophyletic [e.g., *S*. Dublin (Chen et al., 2024)], where the isolates within a serovar cluster in a single lineage and share a common ancestor that is not shared by isolates of any other serovar. Some *Salmonella* serovars have been shown to be paraphyletic [e.g., *S.* Bredeney (Worley et al., 2018)], where the isolates within a serovar cluster in a single lineage and share a common ancestor, but this common ancestor is also shared with isolates of a different serovar. Finally, some *Salmonella* serovars are polyphyletic [e.g., *S.* Kentucky (Chen et al., 2024)], where the isolates within a serovar are spread across multiple divergent lineages and do not share a common ancestor. Multiple divergent lineages of the same *Salmonella* serovar that have emerged independently from each other might present very distinct phenotypic and epidemiological characteristics (Feasey et al., 2016). For example, a recent study analyzing the phylogeny of seven *Salmonella* serovars (i.e., *Salmonella* Cerro, Dublin, Enteritidis, Infantis, Kentucky, Montevideo, and Reading) associated with meat and poultry revealed that polyphyletic lineages of the same serovar might display distinct characteristics, such as two *S.* Reading lineages, one associated with turkey and one associated with swine (Chen et al., 2024).

Although *Salmonella enterica* serovars are highly diverse, they are mainly defined by the combination of 47 somatic (O) and 114 flagellar (H) antigens (McQuiston et al., 2004; Seif et al., 2019). O antigens are lipopolysaccharide (LPS) chains present on the bacterial LPS layer (Liu et al., 2014). The O antigens are encoded by the *rfb* gene cluster (also known as O-antigen gene cluster), which is typically located on the chromosome and consists of 3–20 genes depending on the serogroup. These genes are involved in the synthesis of sugar precursors, sugar transferases and O-unit processing and modification (Liu et al., 2014). H antigens are flagellar proteins subdivided into H1 and H2, encoded by *fliC* and *fljB,* respectively, that contribute to the motility of *Salmonella* (Silverman et al., 1979; Minamino et al., 2018). These flagella genes are alternatively expressed, which means H1 and H2 antigens cannot be expressed at the same time. This phenomenon is mediated by the Hin recombinase (Hughes et al., 1988) and results in phase variation.

Traditional *Salmonella* serotyping (White-Kauffmann-Le Minor scheme; WKL scheme) is based on serological agglutination of the O, H, and, to a lower extent, capsular (Vi) antigens. However, traditional serotyping cannot distinguish between isolates that cluster into distinct lineages of polyphyletic serovars (Wattiau et al., 2011; Achtman et al., 2012; Liu and Hsiao, 2022). Therefore, although traditional serotyping still provides a crucial historical link to epidemiological information, higher resolution in subtyping and characterization of *Salmonella* beyond the serovar level is required for better discrimination. With the advent of whole genome sequencing (WGS) in the last decades, WGS-based subtyping methods enabled researchers and public health agencies to obtain information with higher resolution and faster turnaround time (Ibrahim and Morin, 2018). Importantly, WGS data can also be used for the identification of lineages that independently emerged within a single serovar via phylogenetic analysis using single nucleotide polymorphisms (SNPs) or core genome multilocus sequence typing (cgMLST) (Worley et al., 2018; Yin et al., 2020; Cherchame et al., 2022; Chen et al., 2024).

Previous studies have individually characterized the phylogeny of specific serovars using different methods. For example, Yin et al. (2020) analyzed 4,498 *Salmonella* genomes representing 89 serovars by selecting genomes representing each sequence type (ST) within each serovar and identified seven putative polyphyletic serovars—*Salmonella* Montevideo, Bareilly, Saintpaul, Muenchen, Paratyphi B, Kentucky, and Newport—using MLST for phylogenetic analysis (Yin et al., 2020). Worley et al. (2018) analyzed a set of 445 *Salmonella* genomes representing 260 serovars and found approximately 10% of the serovars to be polyphyletic (Worley et al., 2018). Zhang et al. (2019b) used 2,258 genomes representing 107 serovars, by selecting isolates representing serovar, and found that 24 were polyphyletic using core genome alignment (Zhang et al., 2019b). However, these studies either used a limited number of genomes from each serovar or used a limited number of reference serovars for comparison. Reference serovars are a set of genomes selected to demonstrate the diversity of *Salmonella enterica* subspecies (i.e., *enterica*, *salamae*, *houtenae*, *arizonae*, *diarizonae*, and *indica*) in a phylogenetic analysis. Inclusion of a comprehensive number of these reference serovars in phylogenetic analysis is important as it increases the resolution of the analysis allowing for a higher confidence in the phylogenetic grouping (i.e., mono-, para-, or polyphyly) of the target serovar. In this study, 514 reference genomes representing 301, 49, 127, and 37 serovars of the subspecies *enterica*, *arizonae*, *diarizonae*, and *houtenae* were used, respectively. By analyzing 63,204 genomes across 100 serovars in the present study (92 *Salmonella* serovars) and two previous studies from our group [i.e., 8 *Salmonella* serovars, (Chen et al., 2022; Chen et each ST within each al., 2024)], we aim to bridge these gaps by employing WGS data to (i) delineate the phylogeny of the 100 most common *Salmonella* serovars, (ii) identify the major lineage(s) within polyphyletic serovars, and (iii) to infer antigen divergence between specific serovars and their respective closely-related *Salmonella* serovars. This knowledge will help shift *Salmonella* assessment and control strategies to a phylogeny-based approach, ultimately improving *Salmonella* surveillance and reducing the burden of salmonellosis.

## 2 Materials and methods

### 2.1 Identification of the 100 most common *Salmonella* serovars in NCBI Pathogen Detection

To identify the 100 *Salmonella* serovars most frequently found in NCBI Pathogen Detection (NCBI PD), metadata for all *Salmonella enterica* genomes (*n* = 552,201) available at the time was downloaded, as a CSV file, from the NCBI PD website^1^ on August 8, 2023. *Salmonella* serovar information provided in the “computed type” field in the NCBI PD was used to initially assign isolates to a serotype; isolates without a “computed type” (*n* = 1,409) or isolates where no antigens had been assigned (i.e., the entry was -:-:-) (*n* = 4,741) were identified. Among these isolates, 702 were singletons (i.e., not part of any SNP clusters) and were excluded from downstream analysis since our approach to assign the serovar of an isolate depends on the serotype assignment provided by NCBI PD (see below). Isolates belonging to SNP clusters (*n* = 5,448)—including those lacking computed types (*n* = 1,208) or antigen assignments (*n* = 4,240)—were still accounted for as other isolates in the same SNP clusters could have serotypes assigned by NCBI PD, and the total number of isolates in these SNP clusters were considered. Exclusion of these singletons may have affected the order or the inclusion of some serovars among the 100 most common serovars, as these 702 excluded singletons were not counted. For each serovar, we subsequently determined (i) the total number of isolates assigned to a given serotype, (ii) the total number of SNP clusters for a given serovar, and (iii) the total number of singletons (defined as isolates that do not cluster with any other isolates) found for a given serovar. Serovars were subsequently ranked based on the number of isolates available in the NCBI PD. Among the 100 most common *Salmonella* serovars in the NCBI PD identified with this approach, eight serovars (i.e., *Salmonella* Cerro, Dublin, Enteritidis, Infantis, Kentucky, Montevideo, Reading, and Saintpaul) had been previously investigated by our group using a very similar methodology as the one used here (Chen et al., 2022; Chen et al., 2024).

### 2.2 Retrieval of genome sequences for the most common *Salmonella* serovars

**Figure 1:**
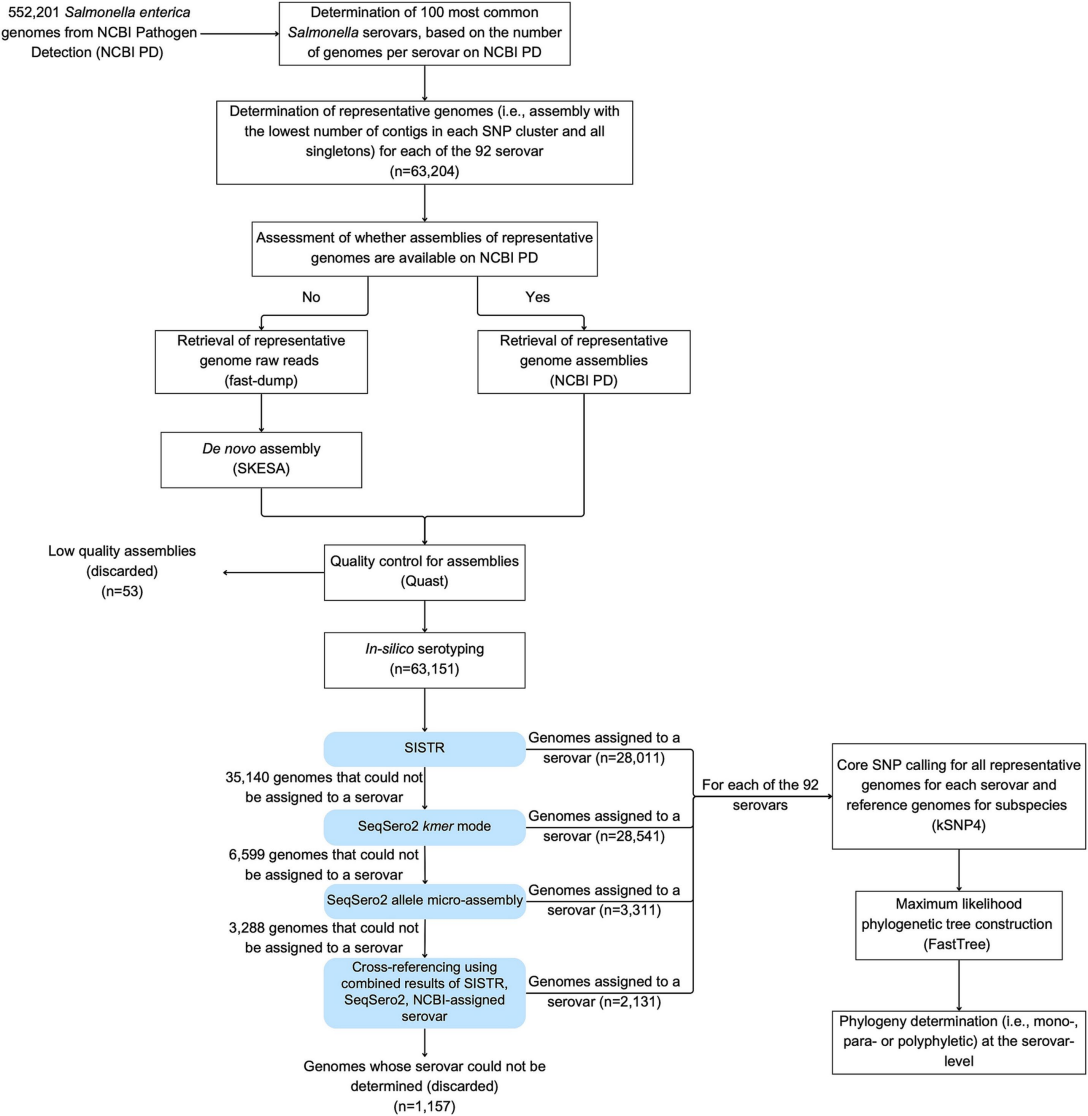
Workflow used in the study to identify the phylogeny of the 100 most common *Salmonella* serovars in NCBI PD. Representative genomes included (i) the assembly with the lowest number of contigs from each SNP cluster (i.e., best assembly), and (ii) assemblies for all singletons. Fifty-three genomes representing singleton isolates were removed because their assemblies were considered with low quality: (i) > 500 contigs, (ii) N50 < 20,000 bp, or (iii) a genome size < 4 or > 6 Mbp. For any genomes where the NCBI-assigned serovar could not be confirmed with SISTR, SeqSero2 *k-mer*, and SeqSero2 allele micro-assembly, serovar confirmation and assignment was carried out by cross-referencing results from multiple approaches. Specifically, in cases where the SISTR results for “qc_status” had warning messages, but the (i) SISTR results for “serovar_antigen” and “serovar_cgMLST,” and (ii) either one or both of the SeqSero2 modes predicted the same serovar, then this serovar was assigned to the genome (even if it differed from the NCBI-assigned serovar).

The workflow followed in Sections 2.2 through 2.4 has been summarized in Figure 1 and the annotated codes used in each pipeline are publicly available in https://github.com/FSL-MQIP/USDA_Salmonella_Phylogeny_Project/tree/main/Codes.

To perform phylogenetic analyses of 92 of the 100 most common serovars that had not been previously analyzed by our group, we identified representative genomes of each of these 92 serovars for subsequent analyses. For each serovar, representative genomes included (i) the assembly with the lowest number of contigs from each SNP cluster, and (ii) assemblies for all singletons. Where available, whole genome assemblies were downloaded from NCBI PD (see text footnote 1); otherwise, raw reads were retrieved using fastq-dump (Chen et al., 2018) and assembled *de novo* using SKESA (Souvorov et al., 2018). QUAST (Gurevich et al., 2013) was then used to assess the quality of each assembly and only assemblies that met the following criteria were used for subsequent analyses: (i) < 500 contigs, (ii) N50 > 20,000 bp, and (iii) a genome size between 4 and 6 Mbp. Singletons not meeting these criteria were not included in further analyses. All assemblies with the lowest number of contigs in a given SNP cluster met the assembly quality criteria.

### 2.3 *In-silico* serotyping

To confirm the serovar assignments obtained from NCBI PD, all representative genomes that passed the quality criteria described above were subjected to *in-silico* serotyping using SISTR (Yoshida et al., 2016). The NCBI-assigned serovar (i.e., “computed type” in the NCBI PD) was considered confirmed if (i) both the antigen prediction (“serovar_antigen”) and the cgMLST prediction (“serovar_cgmlst”) obtained from SISTR matched the NCBI-assigned serovar, and (ii) the SISTR quality control status (“qc_status”) was “PASS.” For genomes that did not meet these criteria, serovar prediction was performed using the *k-mer* approach implemented in SeqSero2 (Zhang et al., 2019a). If the serovars assigned by SeqSero2 *k-mer* and by NCBI matched, the NCBI-assigned serovar was considered confirmed. If the NCBI-assigned serovar still could not be confirmed, serovar prediction was performed using the allele micro-assembly approach implemented in SeqSero2 (Zhang et al., 2019a); if results from SeqSero2 allele micro-assembly matched the NCBI-assigned serovar, the serovar was considered confirmed. For any genomes where the NCBI-assigned serovar could not be confirmed with these steps, the serovar prediction results from all four sources (NCBI, SISTR, SeqSero2 *k-mer*, and SeqSero2 allele micro-assembly) were used for further serovar confirmation and assignment (“serotype assignment by cross referencing”). Specifically, in cases where the SISTR results for “qc_status” had warning messages, but the (i) SISTR results for “serovar_antigen” and “serovar_cgMLST,” and (ii) either one or both of the SeqSero2 modes predicted the same serovar, then this serovar was assigned to the genome (even if it differed from the NCBI-assigned serovar). Singletons that were assigned a different serovar than the serovar assigned by NCBI were removed from any further analyses; traditional serotyping was not carried out to resolve these inconsistencies because isolates were not readily available. When representative genomes from a given SNP cluster were assigned a different serovar than the serovar assigned by NCBI, the second-best assembly in the SNP cluster was then used for *in-silico* serotyping. If the second-best assembly was assigned the same serovar as the one assigned by NCBI, the second-best assembly was kept for downstream analysis.

### 2.4 Maximum-likelihood tree reconstruction

Among the 100 most common serovars, eight serovars (i.e., *Salmonella* Enteritidis, Infantis, Reading, Cerro, Dublin, Kentucky, Montevideo, and Saintpaul), for which our group had previously performed phylogenetic analyses (Chen et al., 2022; Chen et al., 2024), were not included in the phylogenetic reconstruction here, bringing the total number of serovars of interest to 92. To reconstruct the phylogeny of each of the 92 serovars analyzed here, kSNP4 was used to generate a core SNP matrix using a *k-mer* size of 19 nucleotides (Hall and Nisbet, 2023). Each kSNP4 analysis included assemblies for all confirmed representative SNP cluster genomes and singletons, plus 301, 49, 127, and 37 reference genomes representing *Salmonella enterica* subsp. *enterica*, *arizonae*, *diarizonae*, and *houtenae* serovars, respectively (available at: https://github.com/FSL-MQIP/USDA_Salmonella_Phylogeny_Project/tree/main/Reference_Genomes). For *S.* subsp. *enterica*, we used the reference genome set reported by Chen et al. (2024), which included 285 genomes. However, this reference genome set did not include all serovars from the 100 most common serovars identified here (e.g., serovars Derby, Concord). Therefore, the serovars not included in the set reported by Chen et al. (2024) were added, for a total of 301 reference genomes in the set (Chen et al., 2024). For *S.* subsp. *arizonae*, the reference genome set described by Shariat et al. (2021) was used (Shariat et al., 2021). For *S.* subsp. *diarizonae* and *houtenae*, we selected representative genomes from all serovars within these subspecies, based on the isolates available on NCBI PD.

For tree construction, a *S.* subsp. *indica* (GCA_010567565.1), a subsp. *diarizonae* (GCA_010556605.1), a subsp. *arizonae* (GCA_007099465.1), and a *Salmonella bongori* (GCA_037109255.1) genome was used as the outgroup for serovars from subsp. e*nterica*, subsp. *houtenae*, subsp. *diarizonae*, and subsp. *arizonae*, respectively. FastTree was then used to infer the maximum-likelihood phylogeny for each serovar with the General Time-Reversible (GTR)-CAT model and 1,000 bootstrap replicates (Price et al., 2010). The Interactive Tree of Life (iTOL) was used for tree visualization and annotation (Letunic and Bork, 2021).

The phylogenetic grouping definition delineated by Oosterbroek (1987) was used to determine whether a non-monophyletic serovar showed paraphyletic or polyphyletic phylogeny (Oosterbroek, 1987). Briefly, monophyletic serovars are characterized by having a most recent common ancestor (MRCA; the ancestor node of all isolates of a given serovar) that has not given rise to any other serovar. Paraphyletic serovars are characterized by having an MRCA that has given rise to at least another monophyletic serovar or a monophyletic group of other serovars. Polyphyletic serovars are characterized by having an MRCA that has given rise to other serovars that do not form a monophyletic group (Oosterbroek, 1987). Phylogeny for the eight serovars described in previous studies (Chen et al., 2022; Chen et al., 2024) was analyzed again using the criteria described above for consistency. Lineages were identified and labeled alphabetically based on the number of isolates within each lineage. For example, lineage A (e.g., Newport A) represents the lineage with the most isolates within a given serovar, while lineage B represents the lineage with the second largest number of isolates. In this study, a lineage is defined as comprising at least two isolates of the same serovar clustering together. Isolates that do not cluster with any other isolate of the same serovar were defined as “stand-alone singletons” and were not considered as lineages here; these stand-alone singletons were still included in our analyses.

### 2.5 Phylogenetic inference of antigenic variation linked to divergence of closely-related *Salmonella* serovars

To infer the divergence of closely-related serovars and their antigenic formula variations, the five most common serovars available in the NCBI PD database (*Salmonella* Enteritidis, Typhimurium, Newport, I 4,[5],12:i:-, and Infantis) were selected. Two of the five most common serovars analyzed in this section had been previously reported in another study by our group: *S.* Enteritidis and Infantis (Chen et al., 2024). To better infer the phylogeny with a statistically robust framework using a large number of genomes, the maximum likelihood phylogenetic trees for these serovars (i.e., *S.* Enteritidis and *S.* Infantis) were reconstructed using a larger set of reference serovars (*n* = 301; the full list available at: https://github.com/FSL-MQIP/USDA_Salmonella_Phylogeny_Project/tree/main/Reference_Genomes) and core SNPs to determine diversification events of the five most common *Salmonella* serovars. Closely-related serovars were defined as all serovars that (i) share an MRCA with a lineage belonging to at least one of the five serovars analyzed in this section, and (ii) where the MRCA node has a bootstrap value of > 0.7. The antigenic formula of monophyletic and paraphyletic lineages belonging to the five serovars analyzed here were compared to the antigenic formula of their closely-related serovars; for paraphyletic lineages, the antigenic formula of a given lineage was also compared to that of serovars paraphyletically-clustered within the lineage. Lineages that share an MRCA with ≥ 5 serovars (e.g., I 4,[5],12:i:- B and D) were not included in the antigenic formula diversification analysis. Stand-alone singletons were also not included in the antigenic formula diversification analysis as they were not considered lineages. The antigenic differences between the five serovars of interest and their respective closely-related serovars were further classified as somatic (O) or flagellar (H) differences. Differences in O antigens were further divided into (i) major difference (i.e., differences that lead to O antigens that change the serogroup of the serovars) and (ii) minor differences (i.e., differences that lead to O antigens that do not change the serogroup of the serovars). Hence, major O antigen differences involve serovars that belong to different serogroups, while minor O antigen differences involve serovars that belong to the same serogroup. A total of nine categories were used to describe the overall antigen differences as (i) H1 differences only, (ii) H2 differences only, (iii) H1 and H2 differences, (iv) H2 and minor O differences, (v) H2 and major O differences, (vi) H1 and minor O differences, (vii) H1 and major O differences, (viii) H1, H2 and minor O differences, and (ix) H1, H2 and major O differences.

## 3 Results

### 3.1 The 100 most common serovars in the NCBI PD represent 94.55% of the total *Salmonella enterica* genomes in the NCBI PD database

A total of 552,201 genomes downloaded from NCBI PD were used to determine the 100 most common *Salmonella* serovars in this database (based on the serotype assignment captured in the “computed type” field). The 100 most common serovars represented *S. enterica* subspecies *enterica* (*n* = 96), *arizonae* (*n* = 1), *diarizonae* (*n* = 1), and *houtenae* (*n* = 2), and were classified into 23 serogroups that are B (*n* = 21), C_1_ (*n* = 19), C_2_-C_3_ (*n* = 13), D_1_ (*n* = 11), E_1_ (*n* = 8), G (*n* = 7), and 17 serogroups that contained less than five serovars each (*n* = 21). The five most common serovars were, in order, (i) *S.* Enteritidis, (ii) *S.* Typhimurium, (iii) *S*. Newport, (iv) *S*. I 4,[5],12:i:-, and (v) *S.* Infantis (see Supplementary Table 1 for full list). The 100 most common serovars included 93 named serovars as well as seven unnamed serovars (e.g., I 4,[5],12:i:-; the fourth most common serovar). Overall, based on the “computed type” field on NCBI PD, the 100 most common serovars represent 94.55% of all *Salmonella enterica* genomes in the NCBI PD database (Supplementary Figure 1).

### 3.2 *In-silico* serotyping of the representative genomes for the 100 most common *Salmonella* serovars showed that only 1.52% of them do not match the serotype assigned by the NCBI PD

In total, 63,204 representative genomes were initially selected for our analyses based on the NCBI PD’s “computed types” prediction (Table 1). However, 53 genomes were discarded as either their genome data were no longer available on NCBI, or the genome assemblies did not meet the assembly quality criteria. These 53 genomes were singletons, and none of the SNP clusters were discarded during quality control. *In-silico* serotyping approaches, including SISTR, SeqSero2 *k-mer*, and SeqSero2 micro-assembly were used to confirm or revise the NCBI PD serovar assignments for the 63,151 high-quality genomes representing among the 100 most common *Salmonella* serovars (referred as “target serovars” hereon) on NCBI PD. As each genome represent a distinct isolate, we will use the term “isolate” going forward. The 63,151 isolates included (i) 20,497 isolates representing each a SNP cluster and confirmed as belonging to the target serovar, (ii) 41,497 singleton isolates confirmed as the target serovar, and (iii) 1,157 isolates that were not confirmed as the target serovar. The 1,157 isolates that were not confirmed as the target serovar listed on NCBI PD included (i) 957 isolates (1.52%) where the *in-silico* approaches used here identified a different serovar than the serovar assigned on NCBI PD, (ii) 171 isolates (0.27%) for which the genome data were contaminated with multiple serovars as determined by SeqSero2 micro-assembly results, and (iii) 29 isolates (0.05%) where the *in-silico* approaches used here did not identify an O antigen.

**Table 1:** Serovar identification of representative genomes via *in-silico* serotyping tools for the 100 most common *Salmonella* serovars available on NCBI PD.

*In-silico* serotyping assignment steps	Number^a^	Percentage (%)
1. *Representative genomes based on NCBI’s computed types prediction*^b^	*63,204*	*100*
1.1. Analyzed genomes via *in-silico* approaches	63,151	99.92
1.1.1. Genomes representative of SNP clusters that were confirmed as target serovar	20,497	–
1.1.2. Genomes representative of singletons that were confirmed as target serovar	41,497	–
1.1.3. Representative genomes not confirmed as target serovar	1,157	–
1.2. Representative genomes that were not analyzed (e.g., no assemblies available, low assembly quality)	53	0.08
2. *Analyzed genomes using in-silico approaches*	*63,151*	*100*
2.1. Serotype of representative genomes confirmed by SISTR	28,011	44.36
2.2. Serotype of representative genomes confirmed by SeqSero2 *k-mer*-based approach^c^	28,541	45.19
2.3. Serotype of representative genomes confirmed by SeqSero2 micro-assembly mode^d^	3,311	5.24
2.4. Serotype of representative genomes confirmed by cross-referencing^e^	2,131	3.37
2.5. Representative genomes that were confirmed a different computed type than predicted by NCBI	957	1.52
2.6. Representative genomes discarded due to contamination, or not in the WKL Scheme	171	0.27
2.7. Representative genomes for which the somatic (O) antigen was not identified per SeqSero2 micro-assembly mode results	29	0.05

a For detailed data at serovar-level, refer to Supplementary Table 2.

b Computed type on NCBI PD is the antigen formula prediction via SeqSero2.

c Analysis conducted for representative genomes for which their serotype was not confirmed by SISTR.

d Analysis conducted for representative genomes for which their serotype was not confirmed by SISTR or SeqSero2 *k-mer*-based approach.

e For genomes where the NCBI-assigned serovar could not be confirmed with SISTR, SeqSero2 *k-mer* and microassembly, the serovar prediction results from all four sources (NCBI, SISTR, SeqSero2 *k-mer*, and SeqSero2 allele micro-assembly) were used for further serovar confirmation and assignment.

Using SISTR alone, out of the 63,151 representative isolates, the serotype for 28,011 isolates was confirmed. However, we observed that the serotype of 32,552 representative isolates belonging to 43 target serovars could not be confirmed via SISTR (e.g., *Salmonella* Newport, Javiana, and Muenchen; Supplementary Table 2). The SeqSero2 *k-mer* approach confirmed the serotype of 28,541 out of the 35,140 representative isolates not confirmed by SISTR, and the SeqSero2 micro-assembly approach confirmed the serotype of 3,311 out of 6,599 representative isolates that were not confirmed by either SISTR or SeqSero2 *k-mer* approaches. Lastly, cross-referencing was used to confirm the serotype of 2,131 out of 3,288 representative isolates not confirmed by the individual approaches.

Serovars Montevideo, I 4,[5],12:b:-, and IV 48:g,z_51_:- had the largest number of isolates for which the results from our *in-silico* serotyping analyses did not match the NCBI-assigned serovar. Serovar Montevideo (antigenic formula 6,7,14,[54]:g,m,[p],s:[1,2,7]) had 136 isolates that were confirmed a different serovar than the NCBI-assigned serovar (i.e., Montevideo). Based on our *in-silico* analyses, these 136 isolates were either identified as serovar Bareilly (*n* = 25; antigenic formula 6,7,14:y:1,5) or lacked sufficient information for an unambiguous serovar assignment (*n* = 111). Serovar I 4,[5],12:b:- had 114 isolates that did not match the NCBI-assigned serovar (i.e., I 4,[5],12:b:-), these 114 isolates were identified as the serovar Paratyphi B (antigenic formula 1,4,[5],12:b:1,2). Finally, serovar IV 48:g,z_51_:- had 110 isolates that did not match the NCBI-assigned serovar (i.e., IV 48:g,z_51_:-), these isolates were identified as IIIa 48:g,z_51_:-.

### 3.3 Phylogenetic analysis of the 100 most common *Salmonella* serovars showed that 19 are monophyletic, 9 are paraphyletic, and 72 are polyphyletic

Maximum-likelihood trees (Supplementary Figures 2–95, including the trees of the 92 serovars analyzed here, and the re-analyzed trees of the serovars *S.* Enteritidis and *S*. Montevideo) were analyzed at the serovar level to identify serovars that were mono-, poly-, or paraphyletic; further analyses were performed to identify monophyletic and paraphyletic lineages within the 72 polyphyletic serovars. Among the 100 most common *Salmonella* serovars, which include 92 serovars analyzed here (see Supplementary Figures 2–27, 29–64, and 66–95 for the maximum-likelihood phylogenetic trees at https://github.com/FSL-MQIP/USDA_Salmonella_Phylogeny_Project/tree/main/Supplementary_Figures) and 8 serovars analyzed in two previous studies (Chen et al., 2022; Chen et al., 2024), our results showed that (i) 19% of the serovars are monophyletic, (ii) 9% are paraphyletic, and (iii) 72% are polyphyletic (Figure 2); polyphyletic serovars presented either more than one lineage, or one lineage and at least one stand-alone singleton. Across all 100 serovars, we identified 252 lineages (i.e., monophyletic or paraphyletic lineages); the maximum number of lineages was found for serovar Oranienburg, which included 10 lineages. Among polyphyletic serovars with more than one lineage, 215 lineages were identified including (i) 165 monophyletic, and (ii) 50 paraphyletic lineages.

**Figure 2:**
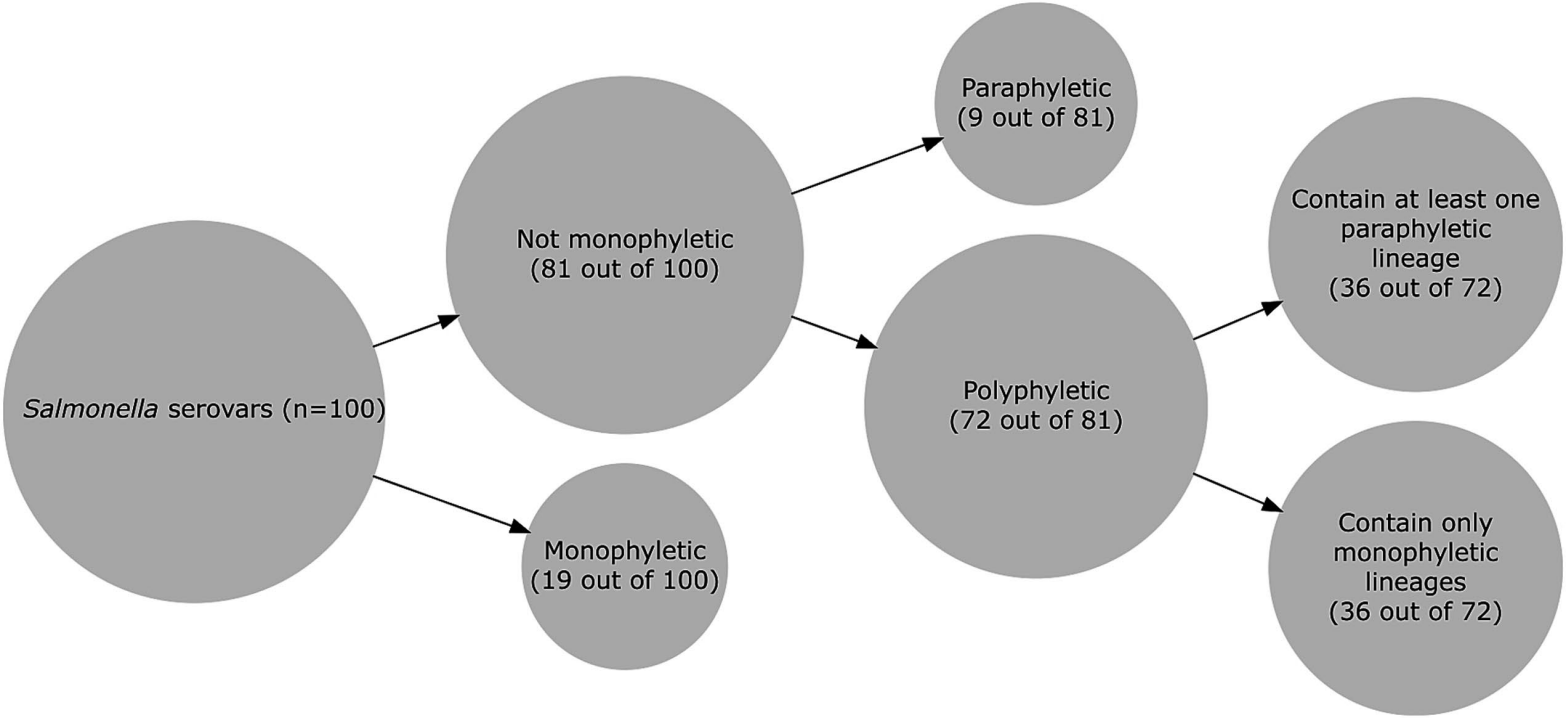
Phylogenetic classification of the 100 most common *Salmonella* serovars available on the NCBI PD database. The size of the circle is proportional to the number of *Salmonella* serovars in a given group.

Among the 19 monophyletic *Salmonella* serovars identified here (see Table 2 for a full list), *Salmonella* Javiana, Typhi, and Braenderup were the three most common monophyletic serovars with 16,637, 13,735, and 9,943 confirmed isolates, respectively. *Salmonella* Haifa was the least common monophyletic serovar with 353 confirmed isolates (Supplementary Table 1).

**Table 2:** Overall phylogenetic grouping (i.e., mono-, para-, or polyphyly) of the 100 most common *Salmonella* serovars on NCBI PD (in alphabetical order).

Phylogenetic grouping	*Salmonella* serovars
Monophyletic	Anatum, Berta, Braenderup, Cotham, Dublin, Haifa, Heidelberg, Inverness, Javiana, Lubbock, Minnesota, Napoli, Norwich, Ohio, Panama, Typhi, Uganda, Weltevreden, I 9:l,z_28_:-
Polyphyletic	Adelaide, Agama, Agona, Alachua, Albany, Altona, Bareilly, Blockley, Bovismorbificans, Brandenburg, Cerro, Chester, Choleraesuis, Coeln, Concord, Corvallis, Cubana, Derby, Eastbourne, Enteritidis, Give, Hadar, Hartford, Havana, Hvittingfoss, Indiana, Infantis, Kedougou, Kentucky, Kiambu, Kottbus, Litchfield, Liverpool, Livingstone, Lomalinda, London, Manhattan, Mbandaka, Meleagridis, Miami, Mikawasima, Mississippi, Montevideo, Muenchen, Muenster, Newport, Oranienburg, Orion, Oslo, Ouakam, Paratyphi A, Paratyphi B, Pomona, Reading, Rissen, Rubislaw, Saintpaul, Sandiego, Schwarzengrund, Senftenberg, Stanley, Tennessee, Thompson, Typhimurium, Virchow, Worthington, I 4,[5],12:i:- (monophasic Typhimurium), I 4,[5],12:b:-, IIIa 41:z_4_,z_23_:-, IIIb 61:k:1,5,(7), IV 48:g,z_51_:-, IV 50:z_4_,z_23_:-
Paraphyletic	Agbeni, Baildon, Bredeney, Carrau, Gaminara, Goldcoast, Johannesburg, Poona, Urbana

Nine of the 100 *Salmonella* serovars analyzed in this study were classified as paraphyletic. These serovars have only one lineage which includes all isolates assigned to the target serovar. However, within each given lineage, we have identified at least one isolate that belongs to a different serovar. For example, serovar Poona has one paraphyletic lineage that includes 3,908 confirmed isolates, as well as serovar Bristol. Serovar Agbeni has 1,851 confirmed isolates clustered within one lineage that also includes serovars Limete and Ituri. Finally, serovar Johannesburg has 1,730 confirmed isolates clustered within one lineage that also includes serovar Urbana.

Overall, among the 72 polyphyletic serovars, 36 serovars contain at least one paraphyletic lineage (e.g., *Salmonella* Enteritidis, Typhimurium), and 27 serovars contain only monophyletic lineages (e.g., *Salmonella* Newport, Kentucky). The remaining nine polyphyletic serovars (e.g., *Salmonella* Agona, Mbandaka, Indiana) include only one monophyletic/paraphyletic lineage and one or two stand-alone singletons; for example, *S*. Agona had 7,810 confirmed isolates, with 7,809 of the isolates clustering in a monophyletic lineage, and one isolate as a stand-alone singleton. *Salmonella* Enteritidis, Typhimurium, Newport, I 4,[5],12:i:-, and Infantis were the most common polyphyletic serovars.

### 3.4 For most polyphyletic serovars, more than 90% of the isolates represent a single lineage

The 72 polyphyletic serovars identified in this study included between one and 10 lineages (Table 3). Polyphyletic serovars with more than one lineage (*n* = 63) most commonly included two, three, or four lineages (25, 13, and 16 serovars, respectively). Among the 63 serovars with more than one lineage, 47 serovars had only one lineage including > 10% of the isolates. Among these 47 serovars, 42 serovars had > 90% of the isolates grouped in a single lineage. For example, *S.* Typhimurium had 99.9% of the isolates included in a single lineage (Typhimurium A; see Table 3). The remaining 16 of the 63 polyphyletic serovars with at least two lineages identified in this study, had multiple lineages with > 10% of the isolates. For example, *S.* Newport had 38,377 isolates distributed in four lineages: Newport A contained 61.6% (*n* = 23,633 isolates), Newport B contained 37.0% (*n* = 14,230 isolates), while the remaining 1.4% (*n* = 514 isolates) were distributed in Newport C and D (Table 3).

**Table 3:** Distribution of the 100 most common *Salmonella* serovars available on NCBI PD by number of lineages and the number of serovars that have one, two, or three lineages containing more than 10% of all genomes within each serovar.

Number of lineages^a^	Number of serovars	Number of serovars that contain					
		Only one lineage with > 10% of isolates	Example^b^ (lineage—% of isolates in a given lineage)	Two lineages that each has > 10% of isolates	Example^b^ (lineage—% of isolates in a given lineage)	Three lineages that each has > 10% of isolates	Example^b^ (lineage—% of isolates in a given lineage)
1^c^	37	37	Javiana—100%	NA		NA	
2	25	21	Infantis A—99.9%^d^	4	Kentucky A—82.7%^d^ Kentucky B—17.2%^d^	NA	
3	13	11	Montevideo A—99.9%^d^	1	Bovismorbificans A—66.2% Bovismorbificans B—33.4%	1	Reading A—74.2%^d^ Reading B—13.2%^d^ Reading C—12.6%^d^
4	16	8	Typhimurium A—99.9%	6	Newport A—61.6% Newport B—37.1%	2	Bareilly A—48.2% Bareilly B—37.6% Bareilly C—14.1%
5	1	1	Cerro A—92.7%	NA		NA	
6	3	3	Adelaide A—88.5%	NA		NA	
7	3	1	Miami A—84.9%	1	Derby A—81.3% Derby B—11.8%	1	Havana A—48.5% Havana B—27.4% Havana C—13.4%
8	1	1	Enteritidis A—99.3%^d^	NA		NA	
10	1	1	Oranienburg A—85.1%	NA		NA	

a Stand-alone singletons were not considered as lineages and are not included in the table.

b The full list of serovars can be found in Supplementary Table 1.

c Among these 37 serovars, 28 are either monophyletic or paraphyletic, while the remaining 9 are polyphyletic serovars, each consisting of one lineage plus stand-alone singleton(s).

d Phylogenetic group information and number of genomes were retrieved from the previous studies conducted by our group (Chen et al., 2022; Chen et al., 2024).

NA, Not applicable.

### 3.5 Closely-related serovars diverged primarily through changes in the H1 antigen

**Figure 3:**
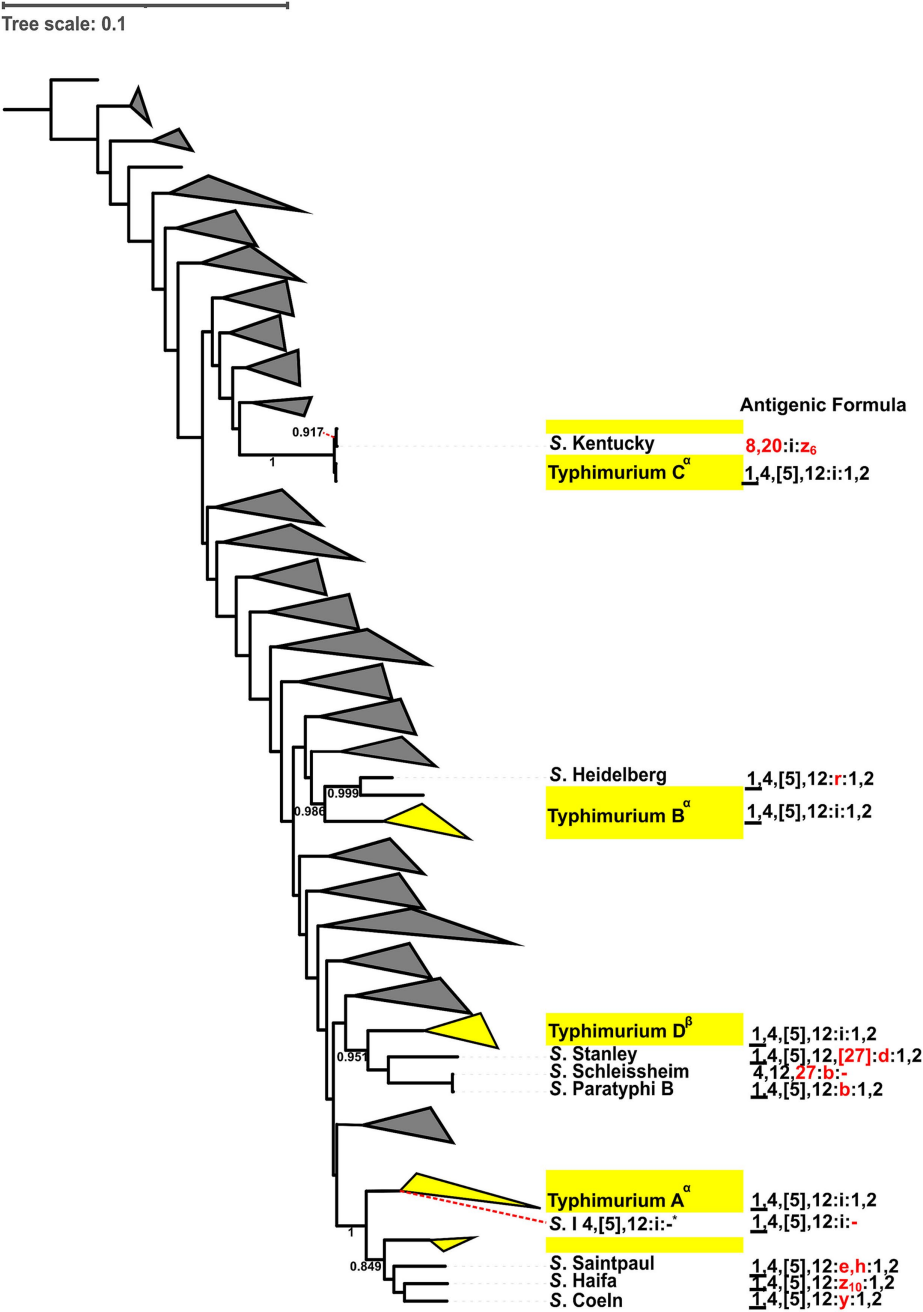
Antigenic formula diversification comparisons of the four lineages of *S.* Typhimurium (i.e., Typhimurium A-D) with corresponding closely-related serovars. Closely-related serovars share the same most recent common ancestor as the *S.* Typhimurium lineages. Differences in somatic (O) and (H) flagellar antigens between closely-related serovars and *S.* Typhimurium lineages are indicated in red. Bootstrap values are provided for the ancestral nodes. Antigenic formula comparisons were made if the bootstrap value of ancestral nodes between Typhimurium lineages and closely-related serovar was >0.7. Closely-related serovars and their antigenic formulas are shown explicitly. Yellow triangles represent genomes from the target serovar (i.e., *S.* Typhimurium), while branches representing non-Typhimurium serovars (distantly related, at least one node away, and not sharing an MRCA) are collapsed to form grey triangles. Superscripts were added to the phylogenetic lineages to differentiate those that are paraphyletic (*α*) from those that are monophyletic (*β*). The *S*. I 4,[5],12:i:- reference isolate (*) clusters within Typhimurium A. Antigenic formulas: (i) underlined O factors (_)—determined by phage conversion and the genome should be lysogenized with the specific converting phage; (ii) curly brackets ({ })—O antigens represented in curly brackets cannot coexist with the other antigens in curly brackets; (iii) square brackets ([ ])—O or H antigens that are present or absent with no relation to phage conversion; (iv) brackets (( ))—O or H antigens that are weakly agglutinable.

To better understand the diversification that ultimately leads to the emergence of different *Salmonella* serovars, we characterized the antigenic variations of the five most common *Salmonella* serovars (i.e., *Salmonella* Enteritidis, Typhimurium, Newport, I 4,[5],12:i:-, and Infantis) and their closely-related serovars (i.e., serovars clustered within paraphyletic lineages or serovars that share an MRCA supported by a > 0.7 bootstrap value). These five serovars included 14 monophyletic and eight paraphyletic lineages. Amongst the 14 monophyletic lineages, five were not compared to any other serovar because they did not share an MRCA with five or less serovars supported by a bootstrap > 0.7. Thus, nine out of the 14 monophyletic lineages were compared to 17 closely-related serovars (e.g., the monophyletic Typhimurium D was compared with *Salmonella* Stanley, Schleissheim, and Paratyphi B as these 4 serovars cluster with a bootstrap value of 0.95, Figure 3). The eight paraphyletic lineages were compared to 16 serovars that fell within the lineages of interest (e.g., the paraphyletic Typhimurium B and *S*. Heidelberg were compared as *S*. Heidelberg clustered within Typhimurium B, Figure 3). Four of these eight paraphyletic lineages were also compared to seven serovars that shared an MRCA supported by a bootstrap value > 0.7 with a given paraphyletic lineages (e.g., paraphyletic lineage I 4,[5],12:i:- A was compared to *Salmonella* Worb and Baildon, Supplementary Figure 98). In total, 40 antigenic diversification events were identified, which included (i) differences in flagellar (H) antigens only (*n* = 13) and (ii) differences in flagellar (H) and somatic (O) antigens (*n* = 27). The differences in flagellar antigens were classified as (i) H1 differences only (*n* = 8), (ii) H2 differences only (*n* = 2), and (iii) H1 and H2 differences (*n* = 3). The differences in flagellar and somatic antigens were classified as (i) H2 and minor O differences (*n* = 2), (ii) H2 and major O differences (*n* = 2), (iii) H1 and minor O differences (*n* = 2), (iv) H1 and major O differences (*n* = 4), (v) H1, H2 and minor O differences (*n* = 6), and (vi) H1, H2 and major O differences (*n* = 11). We identified no diversification events that included only O antigen differences (Tables 4, 5). In summary, of the 40 diversification events we found, 34, 27, and 26 were associated with differences in the H1, O, and H2 antigens, respectively. All 5 serovars assessed here (i.e., Enteritidis, Typhimurium, Newport, I 4,[5],12:i:-, and Infantis) showed similar patterns of diversification, with most events involving the lineage A of a given serovar being associated with variations in flagella antigens only.

**Table 4:** Inference of divergence among the top five most common *Salmonella* serovars available on NCBI PD^a^ by variations in flagellar (H) antigens (*n* = 13).

Types of flagellar and/or somatic antigen variation	Number of serovar divergence by lineage	Serovars involved in divergence events
H1 differences only	8	Enteritidis A: *S.* Gallinarum Enteritidis A: *S*. Dublin Typhimurium A: *S*. Saintpaul Typhimurium A: *S*. Haifa Typhimurium A: *S*. Coeln Typhimurium B: *S*. Heidelberg Typhimurium D: *S*. Stanley Typhimurium D: *S.* Paratyphi B
H2 differences only	2	Typhimurium A: *S*. I 4,[5],12:i:- Infantis A: *S*. Virchow
Both H1 and H2 differences	3	I 4,[5],12:i:- A: *S*. Saintpaul I 4,[5],12:i:- A: *S*. Haifa I 4,[5],12:i:- A: *S*. Coeln

a The five most common *Salmonella* serovars available on NCBI PD: Enteritidis, Typhimurium, Newport, I 4,[5],12:i:-, and Infantis were included in this table. Comparisons with a bootstrap value of their most recent common ancestor less than 0.7 were not analyzed.

**Table 5:** Inference of divergence among the top five most common *Salmonella* serovars available on NCBI PD^a^ by variations in flagellar (H) and somatic (O) antigens^b^ (*n* = 27).

Types of flagellar and/or somatic antigen variation	Number of serovar divergence by lineage	Serovars involved in divergence events
H2 and minor O differences	2	I 4,[5],12:i:- C: *S*. Agama Infantis A: *S*. Colindale
H2 and major O differences	2	Typhimurium C: *S*. Kentucky Newport C: *S*. Braenderup
H1 and minor O differences	2	Newport A: *S*. Litchfield Infantis A: *S*. Oritamerin
H1 and major O differences	4	Enteritidis D: *S*. Schleissheim Enteritidis H: *S*. Apeyeme Enteritidis H: *S*. Fresno Infantis B: *S*. Brijbhumi
H1, H2 and minor O differences	6	Typhimurium D: *S*. Schleissheim Newport B: *S.* Brunei Newport D: *S*. Chailey Newport D: *S*. Hadar Newport D: *S.* Bonariensis Newport D: *S*. Hiduddify
H1, H2 and major O differences	11	Enteritidis D: *S.* Paratyphi B Enteritidis E: *S*. Eboko Enteritidis E: *S*. Uppsala Enteritidis E: *S*. Teddington Enteritidis E: *S*. Weltevreden Enteritidis E: *S*. Stockholm Enteritidis E: *S*. Plymouth Enteritidis F: *S*. Inverness Newport C: *S*. Mikawasima I 4,[5],12:i: - A: *S*. Worb I 4,[5],12:i: - A: *S*. Baildon

a The five most common *Salmonella* serovars available on NCBI PD: Enteritidis, Typhimurium, Newport, I 4,[5],12:i:-, and Infantis were included in this table. Comparisons with a bootstrap value of their most recent common ancestor less than 0.7 were not analyzed.

b Both *S.* Typhimurium and *S*. I 4,[5],12:i:- belong to the O:4 group (B) while *S.* Enteritidis, *S.* Infantis, *S*. Newport are from O:9 (D_1_), O:7 (C_1_) and O:8 (C_2_-C_3_), respectively. O antigen differences were classified as “Major O differences” and “Minor O differences.” Comparisons involving serovars from different serogroups were used to define ‘Major O differences’, while O antigen differences between serovars from the same serogroup were used to define ‘Minor O differences’.

Specifically, out of the 13 diversification events involving five of the eight *S.* Enteritidis lineages, two events (both involving Enteritidis A) involved differences in the H1 antigen only, three events involved differences in the H1 and major O antigens, and eight events involved differences in the H1, H2 and major O antigens (Tables 4, 5 and Supplementary Figure 96). Out of the nine diversification events involving the four *S.* Typhimurium lineages, six events (including three events involving Typhimurium A) involved differences in the H1 antigen only, one event (involving Typhimurium A) involved differences in the H2 antigen only, one event involved differences in the H2 and major O antigens, and one event involved differences in the H1, H2 and minor O antigens (Figure 3; Tables 4, 5). Out of the eight diversification events involving the four *S.* Newport lineages, one event (involving Newport A) involved differences in the H1 and minor O antigens, one event involved differences in the H2 and major O antigens, five events involved differences in the H1, H2 and minor O antigens, and one event involved differences in the H1, H2 and major O antigens (Tables 4, 5 and Supplementary Figure 97). Out of the seven diversification events involving two of the four I 4,[5],12:i:- lineages, one event (involving I 4,[5],12:i:- A) involved differences in the H2 antigen only, three events (all of which involved I 4,[5],12:i:- A) involved differences in the H1 and H2 antigens, one event involved differences in the H2 and minor O antigens, and two events (both involving I 4,[5],12:i:- A) involved differences in the H1, H2 and major O antigens (Supplementary Figure 98 and Tables 4, 5). Out of the four diversification events involving the two Infantis lineages, one event (involving Infantis A) involved differences in the H2 antigen only, one event (involving Infantis A) involved differences in the H2 and minor O antigens, one event (involving Infantis A) involved differences in the H1 and minor O antigens, and one event involved differences in the H1 and major O antigens (Supplementary Figure 99 and Tables 4, 5).

### 3.6 *Salmonella* I 4,[5],12:i:- has emerged multiple times independently from *Salmonella* Typhimurium

**Figure 4:**
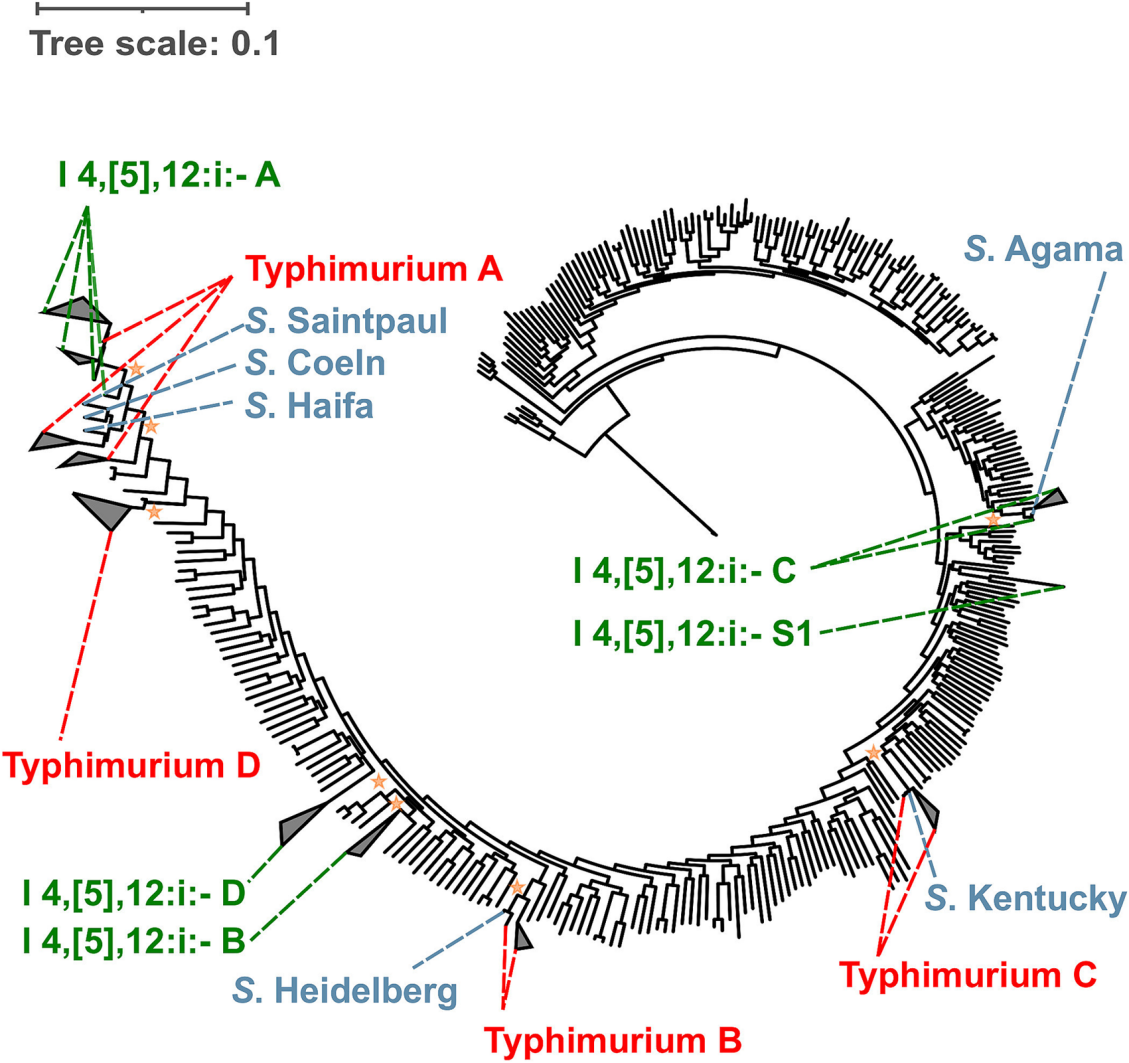
Maximum likelihood phylogenetic tree for *S.* I 4,[5],12:i:- suggests that *S.* I 4,[5],12:i:- has diverged multiple times independently without sharing an MRCA with *S.* Typhimurium. Both *Salmonella* Typhimurium and I 4,[5],12:i:- are polyphyletic serovars. The four lineages belonging to *S.* I 4,[5],12:i:- (i.e., I 4,[5],12:i:- A, B, C, and D) and one stand-alone singleton (i.e., *S.* I 4,[5],12:i:- S1) are shown in green. The four lineages belonging to *S.* Typhimurium (i.e., Typhimurium A, B, C, and D) are shown in red. Reference serovars that clustered within the paraphyletic lineages of *Salmonella* I 4,[5],12:i:- (i.e., I 4,[5],12:i:- A, and C) and Typhimurium (i.e., Typhimurium A, B, and C) are shown in blue. The major I 4,[5],12:i:- lineage (i.e., I 4,[5],12:i:- A) shares an MRCA with *S.* Typhimurium, while the other three *S.* I 4,[5],12:i:- lineages (i.e., I 4,[5],12:i:- B, C, and D) emerged independently without sharing an MRCA with *S.* Typhimurium. In addition to the 301 reference genomes representing *Salmonella* subsp. *enterica* used to reconstruct the phylogenetic trees, reference genomes were selected from Typhimurium A (*n* = 3), B (*n* = 2), C (*n* = 2), and D (*n* = 1) lineages. The average pairwise number of nucleotide substitutions per site was used to report branch lengths. For rooting the tree, one genome belonging to *Salmonella enterica* subsp. *indica* was used as the outgroup. The Shimodaira-Hasegawa test with 1,000 resamples was used to assess the clustering confidence. The orange stars represent the most recent common ancestor of each I 4,[5],12:i:- lineage with a bootstrap value higher than 0.7. For individual phylogenetic trees for *Salmonella* I 4,[5],12:i:- and Typhimurium refer to Supplementary Figures 39 and 90, respectively.

To better understand the evolution of *S.* I 4,[5],12:i:- (often referred to as monophasic Typhimurium) and its relationship to *S.* Typhimurium, a phylogenetic analysis of all representative *S.* I 4,[5],12:i:- isolates and at least one representative *S.* Typhimurium isolate from each *S.* Typhimurium lineage was performed (Figure 4). While I 4,[5],12:i:- lineage A shares an MRCA with *S.* Typhimurium, I 4,[5],12:i:- lineages B, C, and D, and the stand-alone singleton I 4,[5],12:i:- S1 do not. Hence, our results suggest that *S.* I 4,[5],12:i:- has emerged multiple times independently from *S.* Typhimurium.

## 4 Discussion

*Salmonella* subtyping and classification heavily relies on serovar identification. However, previous studies have shown that strains of the same serovar might have different phenotypic characteristics because they belong to lineages that emerged independently from each other. For example, different lineages of *Salmonella* Newport and Kentucky have been suggested to demonstrate varying virulence capabilities, appear to have adapted to different hosts, and appear to exhibit distinct geographic distributions (Cao et al., 2013; Haley et al., 2016). Therefore, identifying distinct lineages within *Salmonella* serovars can aid traceback and outbreak investigations as some of these lineages may show different associations to geographic locations, commodities, and hosts. Although the phylogeny of the most clinically relevant serovars has been well studied (Yin et al., 2020), many other *Salmonella* serovars remain understudied. Hence, this study used the 100 most common *Salmonella* serovars in the NCBI PD database to assess their phylogeny in a standardized manner. With the WGS-based phylogenetic analysis of the 100 most common *Salmonella* serovars in the NCBI PD database, we showed that (i) different *in-silico* serotyping tools (i.e., SISTR, SeqSero2 *k-mer-*based, and SeqSero2 allele micro-assembly approaches) may produce different predictions for some isolates representing certain serovars, (ii) polyphyly is common among *Salmonella* serovars, and (iii) divergence of closely related *Salmonella* serovars is driven primarily by changes in the H1 flagellar antigens, and to a lesser extent by changes in the H2 flagellar and somatic antigens. While our results are consistent with previous studies that showed clustering of *Salmonella* genome sequences by serovar and STs (Cherchame et al., 2022), we showed that (i) a larger proportion of serovars than previously known are polyphyletic; (ii) serovars previously known to be polyphyletic contain more lineages than previously known; and (iii) many serovars include lineages that only have a few isolates with a given serovar. For example, we report for the first time that serovar Hadar, which is commonly found in turkey, is polyphyletic and we also report that serovar Oranienburg include at least 10 distinct lineages [a prior study (Cherchame et al., 2022) only reported 3 lineages for this serovar].

### 4.1 Strategies regarding sampling and WGS data-sharing might bias the geographical representation of *Salmonella* isolates in public databases

The NCBI PD database includes WGS data for more than 550,000 *Salmonella* genomes from across the world, with 57.9% of these genomes originating from the United States (US) (as of August 2023). In the US, regulatory agencies put considerable effort into environmental monitoring of certain food commodities (Crandall et al., 2024) and multiple US federal departments, including but not limited to the US Centers for Disease Control and Prevention (CDC), the US Department of Agriculture (USDA), the Food and Drug Administration (FDA) perform WGS on *Salmonella* isolates and contribute sequence data to NCBI PD. For example, the USDA Food Safety and Inspection Service samples poultry plants several times a year (USDA, 2021), contributing a substantial number of sequence data for US poultry isolates to NCBI PD. Similarly, all human clinical isolates of *Salmonella* obtained in the US are whole genome sequenced and have their WGS data added to NCBI PD. Also, Public Health England (PHE) deposits a substantial number of *Salmonella* genomes sequences in NCBI PD. However, NCBI PD does not reflect the number of reported human cases of *Salmonella* in other countries. For example, the rate of confirmed salmonellosis cases in the Czech Republic (71.9 cases per 100,000 population) and Slovakia (67.5) is high (ECDC, 2024), however, only 21 *Salmonella* isolates from these locations are represented in the NCBI PD database. Therefore, the classification of the 100 most common *Salmonella* serovars used in this study greatly reflects the prevalence of *Salmonella* serovars in the US (and hence may be biased towards serovars with high prevalence in the US), with a few exceptions; for example, *S*. Napoli is among the top 5 serovars causing infection in Italy (Graziani et al., 2015; Sabbatucci et al., 2018), but it is rare in the US. Initiatives, such as that of the Chinese Academy of Science (CAS), which sequenced almost 8,000 *Salmonella* genomes from 22 Chinese provinces, making them publicly available in the Chinese Local *Salmonella* Genome Database version 2 (CLSGDB v2) (Wang et al., 2023b), and also through NCBI, are important to increase the representation of non-US *Salmonella* isolates in NCBI PD.

### 4.2 Different *in-silico* serotyping tools produced different predictions for some isolates of certain serovars

Our findings here are consistent with previous studies that have shown that different *in-silico* serotyping approaches provide results that vary between *in-silico* methods and from those obtained by traditional serotyping (Uelze et al., 2020), as each of these approaches use different databases and algorithms to predict the serotype. It is well known that *in-silico* serotyping is inexpensive and time-efficient compared to traditional serotyping (Banerji et al., 2020). However, constant improvements are still required for robust serotyping. SISTR and SeqSero2 may give ambiguous predictions for some isolates of certain serovars, thus requiring biochemical tests for full characterization when (i) the antigenic formula cannot be fully identified (e.g., missing O antigen genes), and (ii) different serovars share the same antigenic formula in the WKL scheme (e.g., *Salmonella* Choleraesuis, Paratyphi C, and Typhisuis share the same antigenic formula: 6,7:c:1,5). For example, for several serovars, such as *Salmonella* Agona, Enteritidis, and Infantis, Uelze et al. (2020) reported that SeqSero2 was repeatedly unable to identify the O antigen of the analyzed genomes (Uelze et al., 2020). When serovars share the same antigenic formula, identification of phenotypic traits based on genetic markers, if available, could result in more accurate serovar prediction with higher discriminatory power (Yoshida et al., 2016). SeqSero2 already implemented such additional genetic markers (e.g., identifying SNPs in specific genes) for several serovars, like *Salmonella* Enteritidis and Paratyphi B pathotypes. However, implementation of genetic markers for other serovars that share the same antigenic formula (e.g., *Salmonella* Choleraesuis, Paratyphi C, and Typhisuis) would increase the robustness of serovar identification. Lastly, these tools need to be regularly updated with recently identified serovars [e.g., *S.* Lubbock emerged from *S.* Mbandaka by acquiring *fliC* operon of *S.* Montevideo; (Bugarel et al., 2019)] to provide up-to-date serovar identification across serotyping methods. The 1.52% inconsistency between the serotypes assigned by NCBI PD and our approach suggests that the NCBI PD assignment methodology could be improved and that it may be valuable to independently confirm serovar assignments. We observed several isolates from the same NCBI PD SNP cluster (therefore these isolates were all genetically closely related as assessed by SNP differences) that were assigned different serovars. For example, the largest *S.* Enteritidis SNP cluster on NCBI PD includes isolates classified as serovar Hillingdon, and other unnamed serovars (e.g., I 9,46:g,m:1,2) with an antigenic formula different from that of *S.* Enteritidis (i.e., I 1,9,12:g,m:-).

### 4.3 Polyphyly is common among *Salmonella* serovars

Our comprehensive phylogenetic analysis of the 100 most common *Salmonella* serovars, covering 94.55% of the total number of *Salmonella* genomes available in the NCBI PD database, revealed that 72% of the *Salmonella* serovars are polyphyletic. This finding is noteworthy as it contrasts with previous studies that reported different frequencies of polyphyly among *Salmonella* serovars. For instance, Worley et al. (2018) found approximately 10% of *Salmonella* serovars to be polyphyletic, among 445 isolates that represented 260 serovars analyzed in their study (Worley et al., 2018). These authors assessed the phylogeny of *Salmonella* serovars using WGS data of a limited number of isolates from each serovar. Another study used pangenome WGS analysis of 219 genomes and observed polyphyly in 43.1% of the 58 serovars frequently isolated from both the US and Europe (Cherchame et al., 2022). Furthermore, analysis of the 20 most frequently reported serovars associated with human clinical cases in the US suggested that 35% (7/20) of the serovars were polyphyletic based on a 7-gene MLST analysis (Yin et al., 2020). The discrepancy in these findings and the findings reported in the current study highlights the influence of methodological differences in phylogeny inference. The present study represents a robust analysis of the phylogeny of the 100 most common *Salmonella* serovars since we identified genomes to represent each SNP cluster and every singleton within each specific serovar, which resulted in the representation of 63,151 genomes. While previous studies (Yin et al., 2020; Cherchame et al., 2022) showed clustering of *Salmonella* genome sequences by serovar and STs, our analyses included 514 reference genomes representing 301, 49, 127, and 37 serovars of the subspecies *enterica*, *arizonae*, *diarizonae*, and *houtenae*, respectively, in order to identify additional polyphyletic linages; this analysis across a wide range of serovars was needed in order to reliably identify different (polyphyletic) lineages and to identify the closest neighbors of these lineages.

Several factors could lead to differences in the phylogenetic grouping of *Salmonella* serovars. Using an appropriate set of reference serovars to construct phylogenetic trees and including all available isolates of interest during phylogeny construction is crucial. For example, previous studies suggested that *Salmonella* Typhimurium, Enteritidis, Havana, and Montevideo, appeared to be monophyletic (Sangal et al., 2010; Cherchame et al., 2022). However, our results and other studies (D'Alessandro et al., 2018; Zhang et al., 2019b; Yin et al., 2020; Chen et al., 2024) found that these serovars are polyphyletic. With the exception of *S.* Havana, we observed that these serovars have one lineage that includes > 99% of the isolates. This could explain the discrepancy between our results and those from previous studies. Results from previous studies may have reported different findings regarding the phylogeny of these serovars because (i) they selected a limited number of isolates from the target serovar, and (ii) they did not include a large enough number of reference serovars representing subspecies *enterica* to capture the true nature of their phylogeny. This also affects the number of lineages identified in polyphyletic serovars. For example, our analysis of *S.* Oranienburg (using 2,427 Oranienburg genomes, that represent both SNP clusters and singletons) resulted in 10 lineages. However, studies conducted by Cherchame et al. (2022) and Zhang et al. (2019b) using a limited number of representative *S.* Oranienburg isolates only identified three and four lineages, based on 24 and 29 isolates, respectively (Zhang et al., 2019b; Cherchame et al., 2022).

### 4.4 Associations of lineages or clades within *Salmonella* serovars with specific geolocations, commodities, and hosts can aid traceback and outbreak investigations

Our findings revealed that *Salmonella* Typhi is among the most common monophyletic serovars. *S.* Typhi, which causes typhoid fever in humans (Yap and Thong, 2017), shows minimal genetic variation and strong geographical clustering, which can help with epidemiological tracking (Wong et al., 2015; Yap and Thong, 2017). Similarly, *S.* Dublin, another monophyletic serovar primarily associated with cattle, also shows a strong phylogeographical association (Fenske et al., 2019; Kudirkiene et al., 2020).

Several polyphyletic serovars also show association with specific regions at the lineage level. For example, *S.* Newport (Sangal et al., 2010), and *S.* Mississippi (Cheng et al., 2021) have geographically distinct lineages. Specifically, our phylogenetic analysis of *S.* Kentucky revealed two main lineages, and two stand-alone singletons. This is consistent with previous findings that have shown that most *S.* Kentucky isolates fall into two main evolutionary lineages: (i) Kentucky-I (ST152), mostly associated with poultry in the US, and (ii) Kentucky-II (ST198), frequently linked to cattle and human cases in Europe (Tate et al., 2022; Richards et al., 2023). In contrast, *S.* Newport has been linked to many human gastroenteritis cases in the US and Europe (Sangal et al., 2010). According to our analysis, *S.* Newport has four distinct lineages, with two main lineages covering nearly 60 and 37% of the *S.* Newport isolates. Similar to these findings, previous studies suggested that *S.* Newport isolates fall into three distinct lineages, which show geographic patterns; Lineage I (Newport C in this study) was mostly comprised of European isolates, while Lineages II (Newport A in this study) and III (Newport B in this study) were mostly linked to North America (Sangal et al., 2010). *S.* Derby, which our analysis revealed to have seven lineages, was previously reported to show lineage associated host specificities, with isolates in distinct lineages being associated with poultry or swine (Sevellec et al., 2018). In addition, previous studies have also identified STs that are associated with specific reservoirs and human hosts. For example, a Chinese study reported that *S.* Typhimurium ST34 isolates, which are associated with swine, and *S.* Typhimurium ST19 isolates, which are associated with chickens, were found to mainly cause gastro-infection in children and adults, respectively (Wang et al., 2023a). These findings further support that lineage specific characteristics may be valuable in outbreak and traceback investigations.

### 4.5 Divergence of closely related *Salmonella* serovars involves variations in somatic and flagellar antigens, but primarily through changes in the H1 antigen

Our results showed that antigen diversification of the five most common *Salmonella* serovars was primarily associated with differences in the H1 and H2 antigens, compared to differences in the O antigens. This might be related to the complexity of the genetic material underlying the expression of different antigens. H1 and H2 antigens are encoded by single genes, while O antigens are encoded by the *rfb* gene cluster (Silverman and Simon, 1980; Liu et al., 2014; Horvath et al., 2019). Differences in the H1 antigen are likely to be associated with fewer evolutionary changes, ultimately following the principal of parsimony (Kannan and Wheeler, 2012). The O and H antigens are both involved in pathogenesis and are immunogenic, leading to the production of antibodies by the host (Reeves, 1993). The O antigen plays a role in allowing the pathogen to evade phagocytosis by immune cells (Farhana and Khan, 2024). More specifically, variations in the length of the O-antigen chain can obstruct complement-mediated killing and bacterial defenses (Thiriard et al., 2018). It has been suggested that long O-antigen chains are associated with enhanced fitness during *Salmonella*-induced colitis via increased bile resistance (Crawford et al., 2012). On the other hand, Hayashi et al. (2001) concluded that the FliC protein (H1 antigen) will bind to IpaF intracellular receptors and cell surface TLR-5 receptors, which triggers a pro-inflammatory signaling pathway activating the host immune system to remove bacterial infections (Hayashi et al., 2001). Therefore, it is possible that *Salmonella* shows antigen divergence to maintain its ability to colonize hosts, adapt to different environments, or to evade host’s immune response (Liu et al., 2014).

Genomic rearrangement, gene deletion, gene disruption, and horizontal gene transfer followed by homologous recombination are possible mechanisms resulting in emergence of multiple antigenic phenotypes, ultimately leading to divergence of serovars (Vink et al., 2012; Burki et al., 2015; Tanner and Kingsley, 2018; Yin et al., 2020). Previous findings suggest that *S*. I 4,[5],12:i:- emerged multiple times from *S.* Typhimurium (Switt et al., 2009; Arrieta-Gisasola et al., 2020; Wang et al., 2023c). This divergence has been suggested to have occurred primarily through interruptions of the flagellar coding region (Ingle et al., 2021). This genetic alteration typically involves the integration of an insertion sequence into *fljB*, which disrupts the expression of the H2 antigen in *S*. I 4,[5],12:i:- (Arrieta-Gisasola et al., 2020). As a result, *S*. I 4,[5],12:i:- emerged expressing only the H1 antigen (encoded by *fliC*), lacking the ability to switch between two flagellar phases. *S*. I 4,[5],12:i:- is characterized by high multidrug resistance, especially in *mcr*-carrying *S*. I 4,[5],12:i:- ST34, which has been implicated in several outbreaks worldwide (Biswas et al., 2019; Laisnez et al., 2025; Wu et al., 2025). Additionally, the emergence of *S*. I 4,[5],12:i:- ST34 has been associated with increased phage resistance through lysogenic conversion, which may have allowed *S*. I 4,[5],12:i:- ST34 to spread rapidly across multiple host species and become a major concern to public health (Charity et al., 2022). Although it has been clear that *S*. I 4,[5],12:i:- have emerged multiple times, it remained unclear whether all *S*. I 4,[5],12:i:- isolates emerged from *S.* Typhimurium. Our results further increase the understanding of *S*. I 4,[5],12:i:- and *S.* Typhimurium by inferring the overall phylogeny of these serovars and their respective lineages. Noticeably, we observed that only one *S*. I 4,[5],12:i:- lineage shares an MRCA with a *S.* Typhimurium lineage, while the other three *S*. I 4,[5],12:i:- lineages do not, thus suggesting that these three *S*. I 4,[5],12:i:- lineages have emerged independently from *S.* Typhimurium.

Our findings emphasize the importance of using WGS for accurate phylogenetic analysis and lineage identification. While traditional serotype designations are still valuable to maintain a link with historical epidemiological data and to resolve ambiguous *in-silico* results, classification systems (such as serotyping) that heavily relies on phenotypic traits are unlikely to fully capture the genetic diversity and evolutionary relationships within *Salmonella* serovars (Banerji et al., 2020). Serotype-based nomenclature can be useful for monophyletic and paraphyletic serovars, however, for polyphyletic serovars, serotyping does not provide enough discriminatory power to identify different lineages within the serovar. The prevalence of polyphyly observed in our study highlights the limitations of serotyping and the need for genomic approaches to discern lineages of a given serotype. WGS-based phylogenetic analysis provides a more comprehensive understanding of the phylogenetic history of *Salmonella* serovars, which could enable more effective root-cause analysis, as well as traceback and outbreak investigations. Hence, a nomenclature that incorporates both classical serotype information and the phylogenetic lineages (a “hybrid nomenclature”) (e.g., *Salmonella* Kentucky A, *Salmonella* Kentucky B) would be beneficial for researchers, industry, regulators and public health professionals, as it would provide for improved isolate characterization and classification, which can be linked to virulence potential as well as likely sources and geographic origins. While *Salmonella* strain designations based on either solely MLST-derived STs (Achtman et al., 2012) or based on ST and serovar (Chattaway et al., 2021) have previously been proposed, these approaches would not allow stakeholders to easily identify distinct polyphyletic lineages [e.g., as a given phylogenetic lineage may include several STs (Chen et al., 2024)]. Integration of ST into proposed “hybrid nomenclature” would however be easy (e.g., *Salmonella* Kentucky A ST152). Importantly, phylogenetic-based “hybrid nomenclature” is already currently used by the Genome Taxonomy Database (GTDB) project (Parks et al., 2018), in which polyphyletic genera (e.g., *Bacillus*) are named with alphabetical suffixes added to the genus name (e.g., *Bacillus*_A, *Bacillus*_B) to differentiate each polyphyletic lineage, supporting the feasibility of this approach.

## Data Availability

The metadata, reference genomes, codes, and supplementary figures presented in this study can be found in online repositories. Metadata: https://github.com/FSL-MQIP/USDA_Salmonella_Phylogeny_Project/tree/main/Metadata; Reference genomes: https://github.com/FSL-MQIP/USDA_Salmonella_Phylogeny_Project/tree/main/Reference_Genomes; Codes: https://github.com/FSL-MQIP/USDA_Salmonella_Phylogeny_Project/tree/main/Codes; and Supplementary figures: https://github.com/FSL-MQIP/USDA_Salmonella_Phylogeny_Project/tree/main/Supplementary_Figures.

## Footnotes

^1^ https://www.ncbi.nlm.nih.gov/pathogens/
