# Supplementary material for: Phylogeny and divergence of the 100 most common Salmonella serovars available in the NCBI Pathogen Detection database
Source: Front Microbiol. 2025 Jun 13;16:1547190. doi: 10.3389/fmicb.2025.1547190 (PMC12203563; doi:10.3389/fmicb.2025.1547190)
Supplement: Supplementary file 1 [file Data_Sheet_1.zip › Supplementary Figure 1.docx]

Supplementary Material

# Supplementary Figure


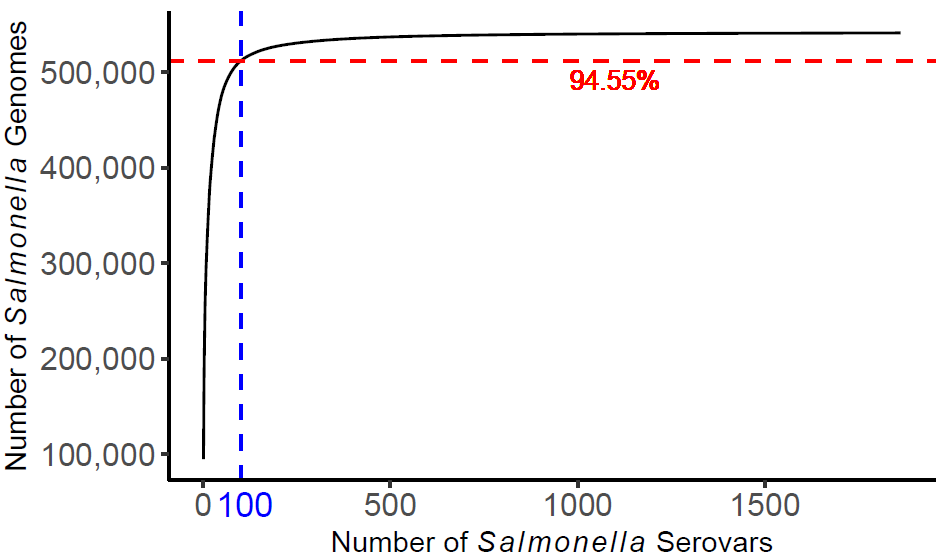


**Supplementary Figure 1.** The number of *Salmonella enterica* genomes present in NCBI PD database by serovars
